# Anti-persister efficacy of colistin and meropenem against uropathogenic *Escherichia coli* is dependent on environmental conditions

**DOI:** 10.1099/mic.0.001403

**Published:** 2023-11-22

**Authors:** Joanna Urbaniec, Maria Getino, Tahnee B-D. McEwan, Martina L. Sanderson-Smith, Johnjoe McFadden, Faisal Hai, Roberto La Ragione, Marwa M. Hassan, Suzie Hingley-Wilson

**Affiliations:** ^1^​ Department of Microbial Sciences, University of Surrey, Guildford, UK; ^2^​ School of Civil, Mining and Environmental Engineering, University of Wollongong, Wollongong, Australia; ^3^​ Molecular Horizons and School of Chemistry and Molecular Bioscience, University of Wollongong, Wollongong, Australia; ^4^​ School of Veterinary Medicine, University of Surrey, Guildford, UK; ^5^​ Department of Infectious Disease, Imperial College London, London, UK

**Keywords:** antibiotic persistence, antimicrobial resistance, *Escherichia coli*, urinary tract infections

## Abstract

Antibiotic persistence is a phenomenon observed when genetically susceptible cells survive long-term exposure to antibiotics. These ‘persisters’ are an intrinsic component of bacterial populations and stem from phenotypic heterogeneity. Persistence to antibiotics is a concern for public health globally, as it increases treatment duration and can contribute to treatment failure. Furthermore, there is a growing array of evidence that persistence is a ‘stepping-stone’ for the development of genetic antimicrobial resistance. Urinary tract infections (UTIs) are a major contributor to antibiotic consumption worldwide, and are known to be both persistent (i.e. affecting the host for a prolonged period) and recurring. Currently, in clinical settings, routine laboratory screening of pathogenic isolates does not determine the presence or the frequency of persister cells. Furthermore, the majority of research undertaken on antibiotic persistence has been done on lab-adapted bacterial strains. In the study presented here, we characterized antibiotic persisters in a panel of clinical uropathogenic *Escherichia coli* isolates collected from hospitals in the UK and Australia. We found that a urine-pH mimicking environment not only induces higher levels of antibiotic persistence to meropenem and colistin than standard laboratory growth conditions, but also results in rapid development of transient colistin resistance, regardless of the genetic resistance profile of the isolate. Furthermore, we provide evidence for the presence of multiple virulence factors involved in stress resistance and biofilm formation in the genomes of these isolates, whose activities have been previously shown to contribute to the formation of persister cells.

## Data Summary

The authors confirm all supporting data and protocols have been provided within the article or through supplementary data files. WGS data are available through NCBI BioProject ID PRJNA805266 (isolates UK 1–4 correspond to SAP1832, SAP1875, SAP1869 and SAP1846 respectively) and NCBI BioProject ID PRJNA993712 (isolates AU 1–3 correspond to MJW2670, MJW2671 and MJW2672 respectively).

## Introduction

Urinary tract infections (UTIs) are common, and are mainly caused by uropathogenic *Escherichia coli* (UPEC) (80 %), followed by *Klebsiella pneumoniae *, *Proteus mirabilis *, *Enterococcus faecalis* and *Staphylococcus saprophyticus* [1]. UTIs disproportionately affect the elderly, where those are predominantly associated with catheter use (CAUTIs), and women due to the shorter length of the female urethra [2]. Those infections, if untreated, can progress to acute kidney infections and/or sepsis, which in turn causes a significant burden on the public health systems. For instance, *E. coli* urosepsis is the most common cause of sepsis in England (48 %), with the 30 day mortality rate reaching 18 % [3].

It is estimated that in the UK one in two women will be treated for a UTI in their lifetime [4], resulting in significant antibiotic consumption. However, approximately one in four women who undergo successful antibiotic treatment will present with a recurring UTI (RUTI) within 6 months of initial diagnosis [5]. Currently, the underlying cause of RUTIs, i.e. re-infection or presence of ‘persister’ cells in the bladder, is poorly understood. Persister cells (or persisters) are a subpopulation of genetically susceptible cells which as a result of their metabolic state become transiently tolerant to a given antibiotic [6]. Persisters have previously been demonstrated to be a crucial factor of treatment failure in pathogens such as *Pseudomonas aeruginosa* [7], *Mycobacterium tuberculosis* [8] and *Staphylococcus aureus* [9]. Several studies have also reported that the majority of RUTIs are caused by an infection with the same UPEC strain [10–12]. This points towards persistence as the underlying cause, and implicates the importance of eradication of persister cells as a prerequisite for successful treatment of UTIs.

In both the UK and Australia, UTIs are routinely treated with nitrofurantoin and trimethoprim-sulfamethoxazole (Bactrim) followed by amoxicillin-clavulanic acid (Augmentin), ciprofloxacin/norfloxacin, pivmecillinam, fosfomycin and cephalexin [4, 13]. However, increasing levels of multidrug resistance are being reported during UTI treatment through large-scale antimicrobial resistance (AMR) surveillance programmes [14, 15]. Therefore, there is likely to be an increasing reliance on ‘last-resort’ antibiotics, such as meropenem and colistin, for the treatment of those infections. Meropenem is a broad-spectrum carbapenem, effective against extended-spectrum *β*-lactamase-expressing strains [16] while colistin is a former veterinary antimicrobial pore-forming peptide that is currently used to treat human infections with multidrug-resistant Gram-negative bacteria [17]. Both aforementioned antibiotics are classified as ‘critical in human medicine’ (category B) by the European Medicines Agency [18]. Since antibiotic persistence is regarded as the ‘stepping stone’ to the development of genetic AMR [19, 20], clinical isolates displaying high levels of persistence to either of the aforementioned antibiotics would be of concern to public health.

In this work, we evaluated the anti-persister activity of meropenem and colistin against a panel of UPEC with varying levels of antibiotic resistance, in both laboratory-standard and urine-pH mimicking environmental conditions. We found high levels of persistence to meropenem amongst all screened UPEC isolates and both environmental conditions, regardless of the antibiotic resistance profile. On the other hand, we found colistin to be significantly more effective than meropenem in eradicating the persister subpopulation in standard laboratory growth conditions, but when these conditions were adapted to mimic the pH of a UTI environment, transient resistance rapidly developed in a subpopulation of cells. To follow up on these observations, we performed whole genome sequencing of our UPEC panel. As expected, we did not find any colistin or meropenem resistance-encoding genes or mutations, confirming the phenotypic nature of the observed survival mechanisms. We also did not find any correlation between multidrug resistance and high antibiotic persistence. However, we recorded multiple virulence genes involved in antibiotic efflux, stress resistance and biofilm formation, whose activities have previously been shown to regulate antibiotic persistence [21].

## Methods

### Strain collection

UPEC isolates were isolated by culture from mid-stream urine samples of hospital patients with an acute UTI, by hospital pathology departments from England (four isolates) and New South Wales, Australia (three isolates), prior to this work (2016–2018; see [22, 23]). Isolates were stored at −80 °C as glycerol stocks [25 % glycerol in Mueller Hinton (MH) broth], and pure cultures were freshly prepared whenever required, as described below.

### Time-kill assays

Bacterial cultures were grown on LB agar (Sigma Aldrich) plates, aerobically at 37 °C for 18–24 h. Single colonies were then selected and incubated in LB Miller (10 g l^–1^ tryptone, 10 g l^–1^ NaCl, 5 g l^–1^ yeast extract) at pH 7.2 (Sigma Aldrich) or M9-glucose minimal medium at pH 6 (10.5 gl^–1^ M9 salts, 0.4 % w/v glucose, 2 mM MgSO_4_, 0.1 % v/v vitamin B1, 100 µM CaCl_2_, 0.05 g l^–1^ FeSO_4_, 0.4 % w/v casamino acids [24], 1 ml in 5 ml flasks)*,* overnight. Incubation was carried out aerobically at 37 °C, with shaking at 200 r.p.m. Overnight (16–18 h) cultures were diluted in LB Miller at pH 7.2 or M9-glucose at pH 6 to an optical density (OD_600_) of 0.05 (starter cultures). Starter cultures were incubated for 3 h at 37 °C in specified media at 200 r.p.m., 1 ml in 5 ml flasks until the cultures reached mid-log phase of growth (OD_600_ of ~0.5 or c.f.u. ml^–1^ of 1×10^8^–3×10^8^ in M9-glucose and OD_600_ of ~1 or c.f.u. ml^–1^ of 5×10^8^–1×10^9^ in LB Miller). OD_600_ measurements, as well as a Miles and Misra (MM) serial dilution series spotted onto oven-dried LB Miller agar plates [25], were performed to determine starting c.f.u. ml^–1^ values (i.e. time 0, prior to antibiotic addition). Next, antibiotics were added at 25× MIC (0.75 µg ml^−1^ meropenem or 25 µg ml^−1^ colistin, determined experimentally) in order to achieve concentration-independent killing, as outlined by a recent consensus statement [6]. Cultures were then returned to the shaking incubator and an MM series was performed at 5 and 24 h following antibiotic addition. At T_5_ and T_24_, the antibiotic was removed by washing prior to carrying out the MM series: 100 µl of sample was removed from the culture at each timepoint and centrifuged at 5000 *g* for 5 min. The cell pellet was then resuspended in 100 µl of appropriate fresh media and this suspension was used in the dilution series. MM plates were incubated aerobically at 37 °C for 18–24 h. Colonies were counted manually with a colony counter. At T_24_, suspected colistin-resistant samples were plated on 25× MIC colistin LB Miller agar plates at specified pH values.

### Antibiotic susceptibility screening

For the determination of the MIC of UK isolates, see the literature [22]. Briefly, bacterial isolates were streaked on MH agar (Sigma Aldrich) plates and incubated aerobically at 37 °C for 16–24 h. Bacterial suspensions were prepared as per the manufacturer’s instructions, by mixing bacterial colonies in deionized water to the density of 0.5 McFarland standard. These were subsequently diluted 1 : 100 into MH broth to a final concentration of 1×10^6^ c.f.u. ml^−1^. Then, 50 µl per well of the cell suspension was then added to Sensitire EU Surveillance *Salmonella */* E. coli* EUVSEC plates (ThermoFisher Scientific). The plates were sealed and incubated at 37 °C for 18 h. The MIC value was determined as the lowest concentration of the antibiotic which inhibited visible bacterial growth (turbidity). In this article, resistance cut-off values were set according to the European Committee on Antibiotic Susceptibility (EUCAST) version 6 MIC breakpoint table [26], to agree with MIC cut-off values applied to Australian isolates.

Antibiotic susceptibility profiles of Australian UPEC isolates were obtained from Ognenovska and colleagues by the disc diffusion assay method [23], with the exception of meropenem and colistin where MIC assays were performed as described above.

For colistin susceptibility, the MIC assay was repeated as above in LB Miller broth at pH 7.2, as well as LB Miller broth at pH adjusted to 6 with HCl.

### Whole genome sequencing (WGS) and bioinformatics analysis

UK isolates have been sequenced prior to this work [22]. Australian isolates were sequenced as a part of this work. Overnight cultures were prepared as described above. DNA was extracted from cultures using a Wizard DNA extraction kit (Promega), according to the manufacturer’s instructions. Illumina short-read genome sequencing (30× coverage) was performed by MicrobesNG Birmingham.

Trimmed reads were assembled with *Shovill* v.1.1.0 [27], modified by T. Seemann) and annotated with *Prokka* v.1.14.6 [28]. Species-level identification was performed based on 16S RNA gene sequences using the Silva 16S RNA database [29]. AMR and virulence genes were identified with *NCBI AMRFinderPlus* v.3.11.11 [30]

### Statistical analysis

All statistical analyses were performed in GraphPad Prism v.8 (GraphPad Software) and results are stated in the respective figure legends.

## Results

### Efficacy of colistin and meropenem against persister cells is dependent on environmental conditions

To evaluate anti-persister activity of colistin and meropenem, time-kill assays were performed in both laboratory-standard rich media (LB Miller broth, pH 7.2) and minimal media (M9-glucose) adjusted to the average pH of human urine (pH 6) [31]. Note that M9-glucose still contains glucose as a carbon source, which is not present in healthy human urine [31], and that growth media composition, in addition to pH, can affect the frequency of persister cells. However, the growth rate of UPEC in this medium was significantly lower than in LB Miller (data not shown), which is more representative of a nutrient-limited environment of a UTI. It should also be considered that bacterial growth can affect extracellular pH through production of various metabolites. In the case of growth in M9 medium, acetate production can decrease culture pH. However, this has been shown to only occur during pre-stationary/stationary phase [32, 33], while mid-exponential phase cultures were used here. For LB Miller, it has been reported to become slightly acidified in the lag/early log phases, and afterwards alkalized to a slightly basic pH (~7.5–8.0) by mid-exponential phase of growth [33].

**Fig. 1.**
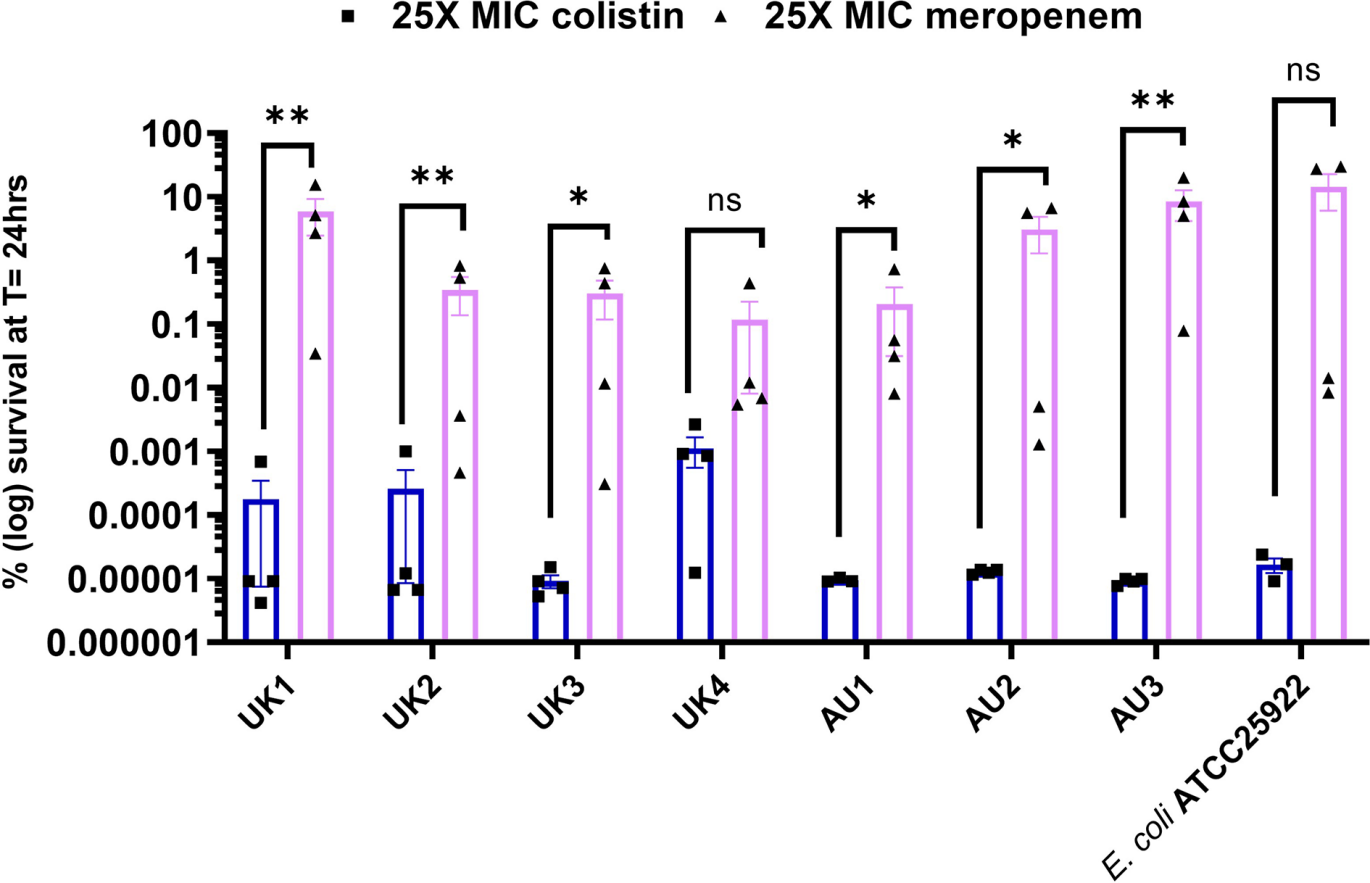
Colistin is significantly more effective than meropenem in eradicating the persister subpopulation in laboratory-standard growth conditions (LB Miller pH 7.2). *E. coli* ATCC25922 was included as a reference strain. *n*=4 from two independent experiments, error bars are sem; limit of detection was 100 c.f.u. ml^−1^; *P<0.05, **P<0.01, and *q*<0.05 by one way ANOVA with Benjamini, Kreuger and Yekutieli false discovery control method.

Time-kill assays in LB Miller pH 7.2 followed the expected bi-phasic killing dynamics (rapid-kill phase of the bulk population followed by a slow/no-kill phase of the persister subpopulation [6]), except UK1 and UK3 where the kill curve shape following exposure to colistin was more characteristic of a susceptible population (rapid-kill phase of the entire population) (Fig. S1, available in the online version of this article). As can be seen in Fig. 1, colistin was significantly more effective in eradicating the persister cells than meropenem. This is probably due to its mechanism of action, as colistin is a membrane pore-forming peptide whose antimicrobial activity is independent of cell growth state, in contrast to the cell wall synthesis inhibitor meropenem [16, 17]. Interestingly, there was no significant difference in persister cell abundance with a given antibiotic between strains, despite the differences in their antibiotic resistance profiles. This suggests that multidrug resistance does not correlate with increased antibiotic persistence.

**Fig. 2.**
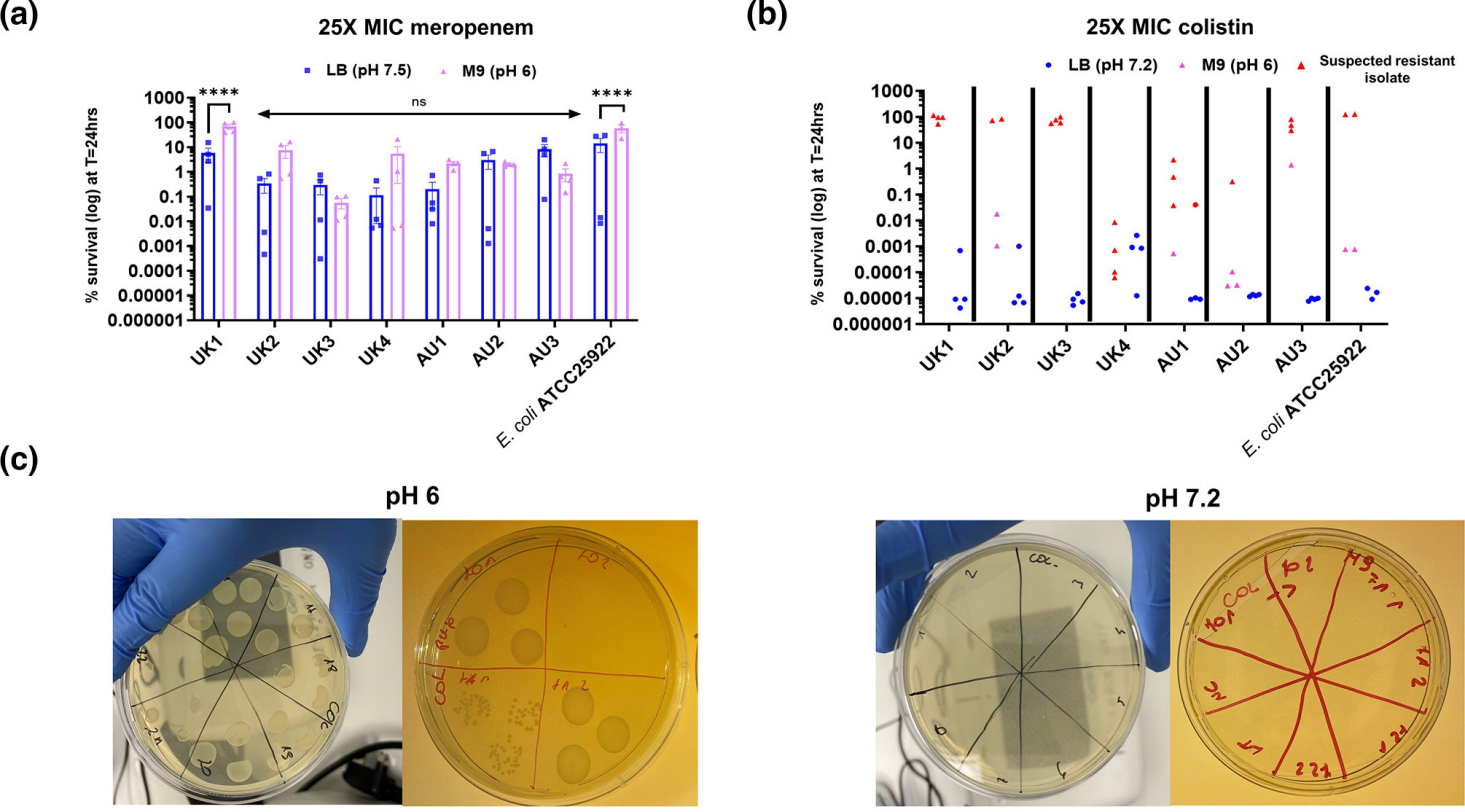
Anti-persister activity of colistin and meropenem is environment-dependent. (a) Survival (%) following 24 h exposure to 25× MIC meropenem in LB Miller (pH 7.2) and M9-glucose media (pH 6). *n*=4 from two independent experiments, error bars are sem; ****P and *q*<0.0001 by two-way ANOVA (antibiotic vs. strain) with Benjamini, Kreuger and Yekutieli false discovery control method. (b) Survival (%) following 24 h exposure to 25× MIC colistin in LB Miller (pH 7.5) and M9-glucose media (pH 6). *n*=4 biological repeats (three technical replicates each), from two independent experiments; red – suspected resistant isolates. (c) Growth of suspected resistant replicates on a 25× MIC colistin agar plate.

Similarly to LB Miller, meropenem time-kill assays in M9-glucose media followed the classical bi-phasic killing dynamics, except for UK1 and *E. coli* ATCC25922 (reference strain) where the kill curve shape resembled a tolerant population (little/no-kill of the entire population within the experimental timeframe) (Fig. S2). The frequency of meropenem persister cells was higher in M9-glucose than in LB Miller, but this difference only reached statistical significance for one of the UPEC isolates (UK1) (Fig. 2a). The observed increased persistence was probably a consequence of a slower growth rate in this medium (data not shown), caused by multiple factors such as altered nutrient availability and low pH, resulting in a reduced susceptibility to meropenem (an antibiotic which inhibits cell wall synthesis and therefore is most active against dividing cells [16]).

Interestingly, although colistin was effective in killing >99.9 % bacterial cells in LB Miller within 24 h of exposure, in M9-glucose at pH 6 after an initial kill phase, ‘resistance’ (i.e. regrowth in the presence of the antibiotic) was observed (Figs 2b and S2). However, when the suspected resistant replicates were plated on 25× MIC colistin agar plates, colonies formed only on plates where the agar pH was adjusted to below 7 (Fig. 2c). It is important to note that no change in the MIC value of colistin was observed when the MIC assay pH was adjusted to 6, and therefore the observed growth cannot simply be explained by an increased colistin MIC at low pH. Colistin is a positively charged peptide which exerts its antimicrobial activity by binding to negatively charged lipopolysaccharides (LPS) on the cell membrane [17]. It is suspected that in the presence of a high concentration of H^+^ ions (i.e. at low pH), the peptide is not able to bind to LPS resulting in transient resistance to colistin rather than a genetic adaptation. This hypothesis was further supported by WGS data, where no colistin resistance genes were found amongst investigated UPEC isolates (Table 1).

**Table 1.** Genetic AMR profile across UPEC genomes Genes were searched for and annotated with NCBI AMRFinder Plus [23].

	**β-Lactams**				**Quinolones**				**Nitrofuran**		
	**Penicillins**		**Cephalos** **-porins**						**Nitrofur** **-atonin**		
	blaEC	blaTEM	blaCTX- M-15	blaEC-5	parE_ S458A	parC_ S80I	gyrA_ D87N	gyrA_ S83L	nfsA_ S33R		
UK1	+	+	+					+	+		
UK2	+										
UK3	+				+	+	+	+			
UK4	+	+									
AU1	+	+		+							
AU2				+							
AU3	+	+				+		+			
	**Folate** **synthesis** **inhibitor**								**Aminogylco** **-side**		
	**Trimetrophim**					**Sulfonamide**			**Streptomycin**		
	dfrA1	dfrA5	dfrA7	dfrA12	dfrA17	sul1	sulB	sulA	aadA1	aadA2	aadA5
UK1			+	+		+		+	+		
UK2											
UK3		+				+		+			
UK4											
AU1						+				+	
AU2	+								+		
AU3					+	+	+				+
							**Macrolide**	**Tetracycline**			
					**Streptothricin**	**Gentamycin**					
			aph(6)-Id	aph(3)-Ib	sat2	aac(3)-IId	mphA	tet(B)	tet(A)		
UK1						+	+	+			
UK2											
UK3											
UK4											
AU1											
AU2					+						
AU3			+	+		+	+		+		
	**Phenicol**	**Phosphonics**		**Multidrug efflux pump**							
	**Chloram** **-phenicol**	**Fosfomi** **-domycin**	**Fosfomycin**								
	catA1	cyaA_ S352T	uhpT_ E350Q	emrD	acrF	mdtM	emrE				
UK1	+			+	+	+	+				
UK2		+		+	+	+					
UK3		+		+	+		+				
UK4				+	+	+	+				
AU1				+	+		+				
AU2			+	+	+		+				
AU3				+	+	+					

### UPEC persistence is independent of multidrug resistance, but could be influenced by expression of virulence factors or antibiotic efflux

Multidrug resistance was a common feature of the investigated UPEC panel with 4/7 isolates (UK1-3 and AU1) classified as multidrug-resistant, i.e. resistant to at least one antibiotic from three or more antibiotic classes (Table 2) [34]. However, all isolates were susceptible to meropenem and colistin by both phenotypic (MIC assay, Table 2) and genotypic (WGS, Table 1) screening. There was no correlation between multidrug resistance and persistence to either meropenem or colistin, as there was no significant difference in the frequency of antibiotic persisters between the investigated isolates (Figs 1 and 2) despite a range of resistance profiles. Interestingly, all investigated UPEC genomes contained the *ariR* (*yagB*) gene encoding biofilm formation and an acid stress resistance regulator [35], and 6/7 isolates also encoded a *Yersinia* pathogenicity island HPI which is involved in resistance to reactive oxygen species (ROS) [36]. Both biofilm formation and ROS exposure have previously been shown to induce formation of persister cells [21]. Finally, genomes of all investigated isolates contained genes encoding multiple multidrug efflux pumps (MEPs) (Table 1) whose overexpression has previously been linked to high antibiotic persistence [21].

**Table 2.** Antibiotic susceptibility of UPEC isolates Yellow signifies resistance, green signifies susceptibility and grey signifies the given antibiotic was not part of the panel. Antibiotics with the same resistance mechanism were grouped. Resistance breakpoints are based on the EUCAST v.6 MIC breakpoint table [26].

	UK1	UK2	UK3	UK4	AU1	AU2	AU3
Amikacin							
Amoxicillin/ampicillin							
Amoxicillin and clavulanic acid							
Cefepime/ceftriaxone							
Cefotaxime							
Ceftazidime							
Cephalexin							
Chloramphenicol							
Ciprofloxacin/norfloxacin							
Colistin							
Gentamycin							
Meropenem/imipenem							
Nalidixic acid							
Nitrofurantoin							
Sulfamethoxazole/sulfafurazole							
Tetracycline							
Tigecycline							
Trimethoprim							

## Discussion

This study investigated anti-persister activity of meropenem and colistin against UPEC. The study demonstrated that persistence to meropenem was high amongst clinical isolates, especially when cells were grown in nutrient-restricted media adjusted to average urine pH. Since meropenem is a cell wall synthesis inhibitor [16], we theorize that in conditions which restrict cell division (e.g. growth in nutrient-limited minimal media or growth *in vivo*), there is a higher frequency of slower growing/non-growing cells resulting in a higher frequency of meropenem persisters. The influence of both population and single-cell growth rates on susceptibility to *β*-lactam antibiotics is a well-known phenomenon [37–40], but our observations have an added clinical implication. Meropenem is prescribed as a treatment for complicated UTI/kidney infections, and relapse has been reported following successful treatment [41], potentially due to the presence of persister cells. UPEC replication *in vivo* is significantly slower that in *in vitro* media [42], which further highlights the importance of clinically relevant models currently not routinely applied in persistence research.

Additionally, we demonstrated that colistin is an effective anti-persister compound, at least in laboratory-standard conditions. However, when UPEC were cultured at acidic pH, we observed a regrowth of the population after 5 h from antibiotic addition. which we subsequently linked to low extracellular pH. An increased MIC of colistin in acidic pH medium has been previously reported in *mcr-1* (mobilized colistin resistance) expressing *E. coli* strains but not in colistin-susceptible strains [41], which is consistent with our observations as we did not observe an MIC change when UPEC isolates were assayed at pH 6. However, our results suggest that in *mcr*-negative *E. coli,* transient colistin resistance can emerge as a subpopulation feature (as the initial rapid-kill phase was still observed) when bacterial cultures are grown at acidic low pH. Although this proposed mechanism requires further experimental validation, we propose that at acidic low pH (high abundance of H^+^ ions), the positively charged colistin peptide is incapable of binding to the bacterial cell membrane in a subpopulation of cells, resulting in emergence of transient resistance which is then lost once the cultures are transferred to a neutral or basic pH environment. This phenomenon appears to be phenotypic rather than genotypic, as it only occurred transiently in a subpopulation of cells in a defined environmental condition (low pH). However, it was also heritable, at least in the timeframe of our experiment. Therefore, we define it as ‘transient resistance’. Heritability suggests potential epigenetic regulation.

From a clinical perspective, this feature of colistin could affect its efficacy in the treatment of UTIs, as the pH of the bladder is generally slightly acidic (~6), although it can fluctuate between 4.5 and 8 depending on diet [31]. On the other hand, it should be considered that during an infection, acidic pH of the bladder can increase due to the replication of urea-digesting pathogens, e.g. *Proteus mirabilis* [43] or certain strains of pathogenic *E. coli* [44] and *Pseudomonas aeruginosa* [45]. This may explain why colistin has been successfully used in the treatment of UTIs [46, 47]. Furthermore, it is noted that in our study we used pH-adapted minimal medium (M9, pH 6) in order to mimic the average pH of healthy human urine [31]. However, as noted previously, the composition of this medium does not reflect the composition of healthy human urine. Notably, M9 medium contains glucose as a carbon source which is absent in the urine of non-diabetic patients. Nutrient source and availability is a known factor influencing persister cell formation [21, 48–50]. Therefore, for future work we recommend addition of time-kill assays performed in artificial urine to better represent a UTI environment, as well as time-kill assays in different growth media in order to investigate if transient colistin resistance spontaneously emerges in any acidic growth conditions or whether additional factors (such as defined ionic concentrations) are required.

Aside from phenotypic observations, we found genes encoding multiple MEPs amongst all investigated UPEC isolates. MEPs are widespread amongst *E. coli* [51]. Yet, to the best of our knowledge, the distribution of MEP-encoding genes amongst UPEC remains unknown as it can differ from lab-adapted *E. coli* strains [52]. Increased antibiotic efflux has been previously shown to increase antibiotic persistence to *β*-lactams in *E. coli* which has been linked to the overexpression of MEP *TolC* [53]. *TolC* can form a tripartite efflux system with *acrF* (AcrEF-TolC), which we found to be present in all investigated UPEC isolates, and whose overexpression was linked to decreased quinolone susceptibility [54]. Our results could point towards a potential involvement of MEP in antibiotic persistence of UPEC; however, this hypothesis would have to be further investigated by looking at the transcriptome prior to and during antibiotic exposure, rather than simply the presence of MEP genes.

Moreover, the presence of genes involved in either biofilm formation or ROS resistance, which were highly prevalent amongst investigated UPEC isolates, could further contribute to the formation of persisters. Biofilms are known to be nutrient-limited and the stringent (nutrient starvation) response is a major pathway of persister cell formation [55]. Indeed, the presence of persisters is thought to be a major contributor to biofilm antibiotic tolerance [48, 50, 56]. On the other hand, it has been previously demonstrated that multiple antibiotic classes damage bacterial cells through ROS production [57]. Therefore, an increased rate of ROS breakdown would increase the chances of antibiotic exposure survival (i.e. persistence). Similarly to the involvement of MEPs in persistence, this hypothesis should be further investigated by looking at the cells’ transcriptome following antibiotic exposure.

Finally, we found no correlation between increased antibiotic resistance and high persistence. It has previously been shown that high persistence can serve as a ‘stepping-stone’ for the development of genetic resistance to antibiotics other than the one which the cell is persistent to, through increased mutation rates and survival probability [19, 58, 59]. Therefore, one could expect a correlation between high levels of persistence and multidrug resistance. However, this ‘co-evolution’ of persistence and resistance would not apply for resistance genes encoded on mobile genetic elements, such as plasmids, which are spread through horizontal gene transfer. Here, we were unable to assign resistance genes to either chromosomal or plasmid origin, and since the majority of resistance genes are encoded on plasmids [60], this would explain the lack of observed correlation.

Overall, this work presents further supporting evidence of the clinical importance of antibiotic persistence in the context of the ‘last-resort’ antibiotics meropenem and colistin, and presents a potential new survival mechanism through which UPEC cells survive colistin exposure.

## Supplementary Data

Supplementary material 1
