# Supplementary material for: Anti-persister efficacy of colistin and meropenem against uropathogenic Escherichia coli is dependent on environmental conditions
Source: Microbiology (Reading). 2023 Nov 22;169(11):001403. doi: 10.1099/mic.0.001403 (PMC10710840; doi:10.1099/mic.0.001403)
Supplement: Supplementary material 1 [file mic-169-1403-s001.pdf]

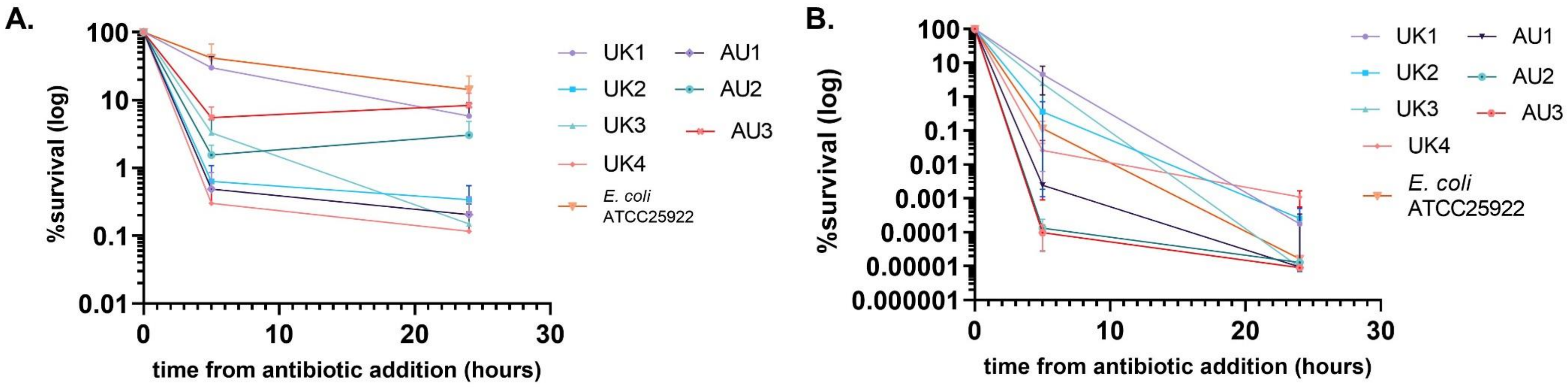

Figure S1: Time-kill assay following exposure to A.25X MIC meropenem and B. 25X MIC colistin in LB Miller (pH 7.2). n=4 from two independent experiments, except UK3 meropenem and AU1 colistin where n=3 due to exclusion a resistant replicate (confirmed by plating on respective antibiotic agar); error bars are SEM. Limit of detection was 100 CFU/mL. **A.** $q < 0.05$ by two-way ANOVA (strain vs. time) with Benjamini, Kruger and Yekutieli false discovery control method for t=0h vs. t=5h for all strains and $q > 0.05$ for t=5h vs. t=24h for all strains. **B.** $q < 0.05$ by two-way ANOVA (strain vs. time) with Benjamini, Kruger and Yekutieli false discovery control method for t=0h vs. t=5h for all strains and $q < 0.05$ for AU2 & AU3 and $q > 0.05$ for UK1-UK4 & AU1 for t=5h vs. t=24h.

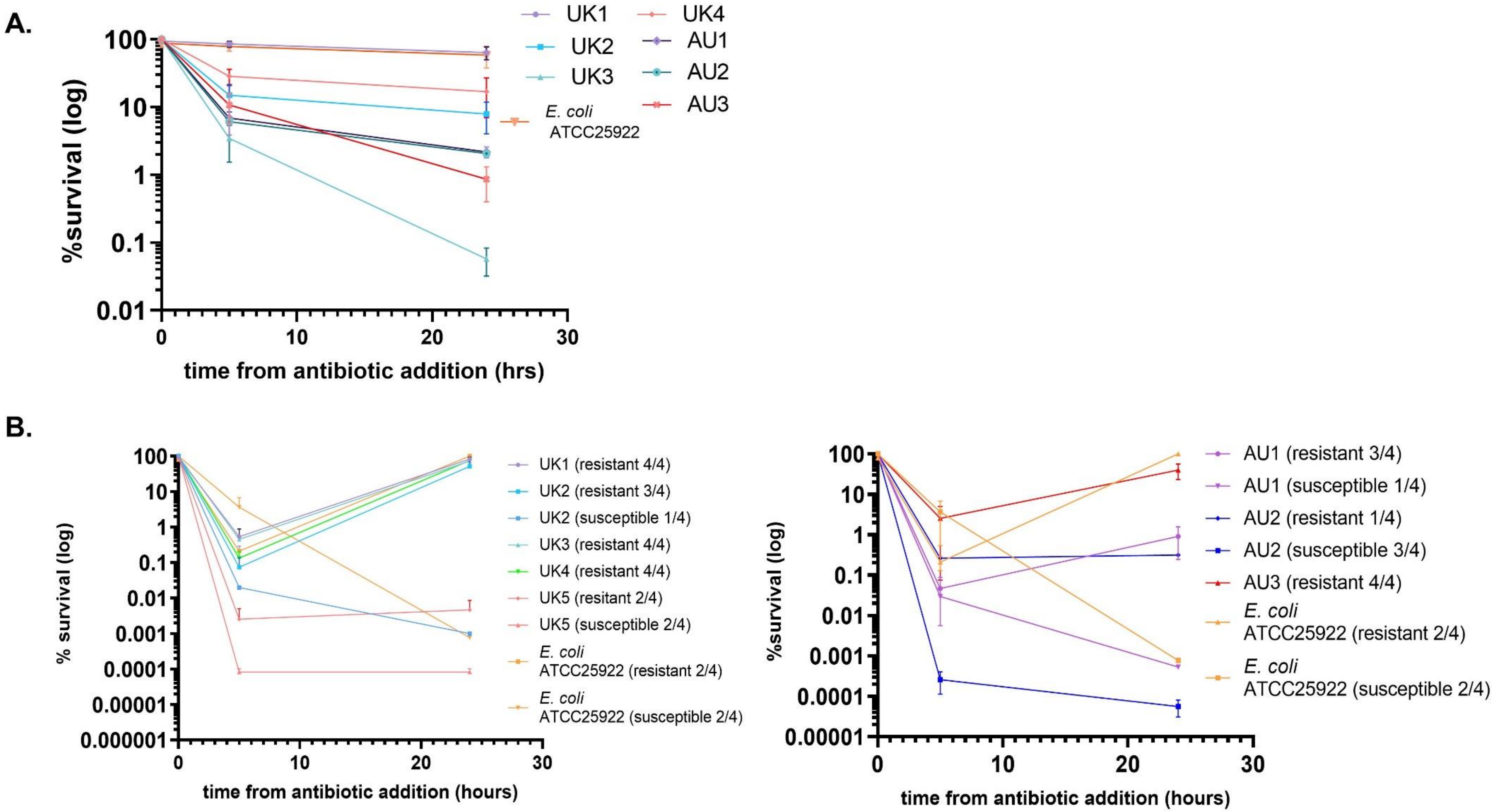

Figure S2: Time-kill assay following exposure to A.25X MIC meropenem and B. 25X MIC colistin in M9-Glucose media (pH 6). n=4 from two independent experiments; error bars are SEM. Limit of detection was 100 CFU/mL. **A.** $q < 0.05$ by two-way ANOVA (strain vs. time) with Benjamini, Kruger

and Yekutieli false discovery control method for t=0h vs. t=5h for UK2 – UK4 & AU1-AU3 and $q < 0.05$ for UK1 & ATCC25922; $q < 0.05$ for t=5h vs. t=24h for UK4 & AU3 and $q > 0.05$ for UK1-UK3 & AU1-AU2.
